# Supplementary material for: Historical Mitogenomic Diversity and Population Structuring of Southern Hemisphere Fin Whales
Source: Genes (Basel). 2023 May 3;14(5):1038. doi: 10.3390/genes14051038 (PMC10218396; doi:10.3390/genes14051038)
Supplement: Supplementary file 1 [file genes-14-01038-s001.zip › genes-2348025-supplementary.pdf]

SUPPLEMENTARY MATERIAL FOR MANUSCRIPT: HISTORICAL MITOGENOMIC DIVERSITY AND POPULATION STRUCTURING OF SOUTHERN HEMISPHERE FIN WHALES

DANIELLE L. BUSS, LANE M. ATMORE, MARIA ZICOS, WILLIAM P. GOODALL-COPESTAKE, SELINA BRACE, FREDERICK I. ARCHER, C. SCOTT BAKER, IAN BARNES, EMMA L. CARROLL, TOM HART, ANDREW KITCHENER, RICHARD SABIN, ANGIE SREMBA, CAROLINE R. WEIR, JENNIFER A. JACKSON

Supplementary Table S1 Metadata for mitogenomes obtained from historical whalebones and baleen of fin whale, *Balaenoptera physalus,* using next-generation sequencing (*n*=34).

#	SampleName	Tissue	Location	Sample Store	No. aligned	Coverage	Mean read depth	STD read depth	Min	Max	FullAlignment	Partial Alignment	Endongenous content
1	SI027	Bone	South Orkneys	BAS specimen store	3090	100	15.1	1.3	13	17	TRUE	TRUE	28.0 %
2	BH01	Baleen	South Georgia	Bob Headland private collection	1300	99.94	7.9	0.9	0	9	FALSE	TRUE	13.1 %
3	NHMUK ZD.2023.9	Bone	South Africa	Natural history museum, London	5554	100	25.3	1	24	26	TRUE	TRUE	54.9 %
4	DCO004	Bone	South Shetlands	BAS specimen store	2302	100	9.9	1.1	9	12	TRUE	TRUE	52.9 %
5	DCO019	Bone	South Shetlands	BAS specimen store	3084	99.9	14.2	4.7	0	32	TRUE	TRUE	1.6 %
6	SI041	Bone	South Orkneys	BAS specimen store	2279	99.98	10.5	4.2	0	29	FALSE	TRUE	19.0 %
7	FKB14	Bone	Falkland Islands	BAS specimen store	337	81.1	1.5	1.1	0	7	FALSE	FALSE	6.8 %
8	FKPT	Bone	Falkland Islands	BAS specimen store	444	88.2	2	1.3	0	7	FALSE	FALSE	3.6 %
9	DCO36	Bone	South Shetlands	BAS specimen store	2972	99.99	13.7	4.8	1	31	FALSE	TRUE	34.5 %
10	BLJS01	Baleen	South Shetlands	John Smellie private collection	2588	99.99	12.5	0.5	2	13	FALSE	TRUE	45.1 %
11	SI025A*	Bone	South Orkneys	BAS specimen store	633	97.7	3	1.9	0	11	FALSE	FALSE	6.9 %
12	SI026	Bone	South Orkneys	BAS specimen store	973	99.4	4.5	2.6	0	14	FALSE	FALSE	8.3 %
13	DCO11	Bone	South Shetlands	BAS specimen store	7190	100	28.1	0.8	27	29	TRUE	TRUE	50.5 %
14	SI038	Bone	South Orkneys	BAS specimen store	2156	100	10.7	3.4	2	24	TRUE	TRUE	5.2 %
15	NHMUK ZD.2023.3	Bone	63'06' S 82'11' E (Area III)	Natural history museum, London	5167	100	24.2	5.5	9	41	TRUE	TRUE	37.2 %
16	FKB38	Bone	Falkland Islands	BAS specimen store	1109	99.79	4	0.7	0	4	FALSE	FALSE	9.2 %

17	NHMUK ZD 1847-2-25-1	Bone	United kingdom	Natural history museum, London	3081	99.99	9	0	9	9	FALSE	TRUE	1.7 %
18	NMS Z.1991.83.2	Bone	South Shetlands	National museum of Scotland, Edinburgh	5187	100	32.4	1.4	30	34	TRUE	TRUE	41.5 %
19	GO146	Bone	South Georgia	BAS specimen store	1776	99.96	7.6	2.6	0	20	FALSE	TRUE	57.7 %
20	BALL01	Baleen	South Georgia	Bob Headland private collection	3266	99.98	15	5.4	0	37	TRUE	TRUE	4.9 %
21	DCO40	Bone	South Shetlands	BAS specimen store	8438	100	37.3	1	36	40	TRUE	TRUE	17.0 %
22	SI034	Bone	South Orkneys	BAS specimen store	2541	99.99	18.9	0.3	18	19	FALSE	TRUE	5.4 %
23	SI046	Bone	South Orkneys	BAS specimen store	817	99.4	3.8	2.4	0	14	FALSE	FALSE	5.4 %
24	DCO27	Bone	South Shetlands	BAS specimen store	3778	100	15.7	0.8	15	17	TRUE	TRUE	26.3 %
25	DCO37	Bone	South Shetlands	BAS specimen store	4385	99.9	20.1	5.7	0	37	TRUE	TRUE	31.4 %
26	FKB16	Bone	Falkland Islands	BAS specimen store	946	99.8	3.1	2.2	0	12	FALSE	FALSE	3.1 %
27	FKB3	Bone	Falkland Islands	BAS specimen store	14428	99.99	66.8	11.9	0	112	TRUE	TRUE	46.7%
28	FKPD	Bone	Falkland Islands	BAS specimen store	16188	100	57.8	2.9	53	62	TRUE	TRUE	14.4 %
29	GO002	Bone	South Georgia	BAS specimen store	7457	100	30.8	0.8	30	32	TRUE	TRUE	16.2 %
30	SI042	Bone	South Orkneys	BAS specimen store	10310	100	47.5	1.8	45	50	TRUE	TRUE	8.7 %
31	DCO20	Bone	South Shetlands	BAS specimen store	57902	100	224.5	2.2	219	227	TRUE	TRUE	51.0 %
32	DCO013	Bone	South Shetlands	BAS specimen store	5612	100	21.7	0.5	21	22	TRUE	TRUE	27.9 %
33	SI021	Bone	South Orkneys	BAS specimen store	2126	100	10	0.1	10	11	FALSE	TRUE	18.2 %
34	SI014	Bone	South Orkneys	BAS specimen store	1912	99.9	8.8	3.1	0	23	FALSE	TRUE	16.9 %

*This sample had many instances of multiple nucleotides at the same base with a read depth of 3 or more and therefore was potentially contaminated and removed

Supplementary Table S2 Metadata for *mtDNA CR* obtained from historical whalebones and baleen of fin whale, *Balaenoptera physalus,* using sanger PCR sequencing (*n*=50). If *mito* = TRUE; mitogenome data also available.

#	SampleName	Tissue	Collection site	Storage location	Ocean basin	mito
1	SI027	Bone	South Orkneys	BAS specimen store	SA	TRUE
2	BH01	Baleen	South Georgia	Bob Headland private collection	SA	TRUE

3	NHMUK ZD.2023.9	Bone	South Africa	Natural history museum, London	SA	TRUE
4	DCO004	Bone	South Shetlands	BAS specimen store	SP	TRUE
5	DCO019	Bone	South Shetlands	BAS specimen store	SP	TRUE
6	SI041	Bone	South Orkneys	BAS specimen store	SA	TRUE
7	FKB14	Bone	Falkland Islands	BAS specimen store	SA	FALSE
8	FKPT	Bone	Falkland Islands	BAS specimen store	SA	FALSE
9	DCO36	Bone	South Shetlands	BAS specimen store	SP	TRUE
10	BLJS01	Baleen	South Shetlands	John Smellie private collection	SP	TRUE
11	SI025A	Bone	South Orkneys	BAS specimen store	SA	FALSE
12	SI026	Bone	South Orkneys	BAS specimen store	SA	FALSE
13	DCO11	Bone	South Shetlands	BAS specimen store	SP	TRUE
14	SI038	Bone	South Orkneys	BAS specimen store	SA	TRUE
15	NHMUK ZD.2023.3	Bone	63'06'S 82'11'E	Natural history museum, London	SA	TRUE
16	FKB38	Bone	Falkland Islands	BAS specimen store	SA	FALSE
17	NHMUK ZD 1847-2-25-1	Bone	United kingdom	Natural history museum, London	NA	TRUE
18	NMS Z.1991.83.2	Bone	South Shetlands	National museum of Scotland, Edinburgh	SP	TRUE
19	GO146	Bone	South Georgia	BAS specimen store	SA	TRUE
20	BALL01	Baleen	South Georgia	Bob Headland private collection	SA	TRUE
21	DCO40	Bone	South Shetlands	BAS specimen store	SP	TRUE
22	SI034	Bone	South Orkneys	BAS specimen store	SA	TRUE
23	SI046	Bone	South Orkneys	BAS specimen store	SA	FALSE
24	DCO27	Bone	South Shetlands	BAS specimen store	SP	TRUE
25	DCO37	Bone	South Shetlands	BAS specimen store	SP	TRUE
26	FKB16	Bone	Falkland Islands	BAS specimen store	SA	FALSE
27	FKB3	Bone	Falkland Islands	BAS specimen store	SA	TRUE
28	FKPD	Bone	Falkland Islands	BAS specimen store	SA	TRUE
29	GO002	Bone	South Georgia	BAS specimen store	SA	TRUE

30	SI042	Bone	South Orkneys	BAS specimen store	SA	TRUE
31	DCO20	Bone	South Shetlands	BAS specimen store	SP	TRUE
32	DCO013	Bone	South Shetlands	BAS specimen store	SP	TRUE
33	SI021	Bone	South Orkneys	BAS specimen store	SA	TRUE
34	SI014	Bone	South Orkneys	BAS specimen store	SA	TRUE
35	WBF02	Bone	South Shetlands	BAS specimen store	SP	FALSE
36	SI048	Bone	South Orkneys	BAS specimen store	SA	FALSE
37	SI047	Bone	South Orkneys	BAS specimen store	SA	FALSE
38	SI046	Bone	South Orkneys	BAS specimen store	SA	FALSE
39	SI044	Bone	South Orkneys	BAS specimen store	SA	FALSE
40	NHMUK ZD 1910-9-24-1	Bone	Ireland	BAS specimen store	NA	FALSE
41	SI040	Bone	South Orkneys	BAS specimen store	SA	FALSE
42	SI031	Bone	South Orkneys	BAS specimen store	SA	FALSE
43	SI005	Bone	South Orkneys	BAS specimen store	SA	FALSE
44	SI003	Bone	South Orkneys	BAS specimen store	SA	FALSE
45	SI002	Bone	South Orkneys	BAS specimen store	SA	FALSE
46	SI001	Bone	South Orkneys	BAS specimen store	SA	FALSE
47	KEC7	Bone	South Georgia	BAS specimen store	SA	FALSE
48	FKPEE	Bone	Falkland Islands	BAS specimen store	SA	FALSE
49	FKB84	Bone	Falkland Islands	BAS specimen store	SA	FALSE
50	FKB17	Bone	Falkland Islands	BAS specimen store	SA	FALSE

Supplementary Table S3 PCR primer sequences and cycler conditions used for amplification of historical fin whale mitochondrial control region (*mtDNA CR*) sequences.

Forward primer (seq)	Reverse primer (seq)	Nucleotide base pairs	Source of primer seq.
Tprowhale 5'TCACCCAAAGCTG RARTTCTA 3'	Dlp8G 5' GGAGTACTATG TCCTGTAACCA 3'	800	Baker et al. 1998; Garrigue et al. 2004
Tprowhale 5'TCACCCAAAGCTG RARTTCTA 3'	Dlp5 5'CCATCGWGATGTCT TATTTAAGRGGAA 3'	500	Baker et al. 1998
Tprowhale 5'TCACCCAAAGCTG RARTTCTA 3'	Dlp4 5'GCGGGWTRYTGRT TTCACG 3'	300	Baker et al. 1998; Ross et al. 2003
PCR amplifications were conducted in 20uL reactions containing 1.1 x QSolution (Qiagen), 0.25mM dNTP mix, 1uM of each primer, 1 x buffer (4mM MgCL2, Takara), 1.5mM additional MgCL2 (Qiagen), 0.15 u/uL Takara hot start TAQ and 1.5uL of gDNA extract. PCR conditions: denaturation at 94'C for 240 s; 38 cycles of 94'C for 45 s, 56'C for 45 s and 72'C for 60 s. Final extension of 72'C for 10 minutes.			

Supplementary Table S4 Best model of evolution for mitogenome partitions determined using PartitionFinder2 for historic (n=27; this study) and contemporary (n=154; Archer et al. 2013) fin whale mitogenomes.

Subset	Partitions/Genes	Best model from Partition Finder 2
1	DLoop_all	GTR+I+G
2	16S_rRNA, all tRNAs	GTR+I+G
3	ND1_pos1, 12S_rRNA, ND4L_pos1, CYTB_pos1, ND5_pos1	TRN+I+G
4	ND2_pos2, ND4L_pos2, ND4_pos2, ND1_pos2	TVM+I+G
5	ND4_pos3, ATP8_pos3, ND2_pos3, ND1_pos3	TRN+I
6	ND2_pos1, ND6_pos2	TIM+I
7	COXI_pos1	TRN+I
8	COXIII_pos2, COXI_pos2, ND3_pos2, CYTB_pos2	HKY
9	COXI_pos3, ATP6_pos3	TIM+G
10	COXIII_pos1, COXII_pos1, ND3_pos1	TIMEF+I

11	ATP6_pos2, ND5_pos2, COXII_pos2	TRN+I
12	ND3_pos3, ND6_pos1, COXII_pos3	TRN
13	ATP8_pos2, ND6_pos3, ATP8_pos1	TRN+G
14	ND4_pos1, ATP6_pos1	TRN+G
15	ND4L_pos3, CYTB_pos3, ND5_pos3, COXIII_pos3	TRN+G

Supplementary Table S5 Pairwise genetic differentiation oceanic comparisons of fin whale populations between ocean basins. φ_{ST} estimates using using *mtDNA CR* sequences (241bp) are shown below the diagonal. *p*-values denoting significance are shown in parentheses.

**Phi-st (below diagonal) and F-st (above diagonal).**					
	North Atlantic (without Mediterranean)	Mediterranean	North Pacific (without Gulf of Californi)	Gulf of California	Southern Hemisphere (circumpolar)
North Atlantic (without Mediterranean)					
Mediterranean	0.03 (<0.001)				
North Pacific (without Gulf of Californi)	0.09 (<0.001)	0.02 (<0.001)			
Gulf of California	0.29 (<0.001)	0.33 (<0.001)	0.18 (<0.001)		
Southern Hemisphere (circumpolar)	0.28 (<0.001)	0.41 (<0.001)	0.53 (<0.001)	0.77 (<0.001)	

Supplementary Table S6 A) Estimates of SHFW haplotype, nucleotide diversity, Tajima's D and Fu's F calculated using *mtDNA CR* sequences of historical fin whale samples from the SH. Sequence length used in this study (bp). Number of polymorphic sites unique to a given region are shown within { }. Standard deviations of diversity estimates are shown in parentheses. *p*-values are shown in square brackets [*p*]. **B)** Pairwise genetic differentiation oceanic comparison of historical SHFW fin whale populations between the South Pacific and South Atlantic. φ_{ST} estimates using using *mtDNA CR* sequences (365bp) are shown below the diagonal. Differences in haplotype frequencies (Fst) are shown above the diagonal. *p*-values denoting significance are shown in parentheses.

**Dataset**	**bp**	**Model of evolution**	**Source**	**No. samples**	**No. unique haplotypes**	**Polymorphic sites {unique to region}**	**Haplotype diversity**	**Nucleotide diversity**	**Tajima's D**	**Fu's F**
**A) Genetic diversity estimates using mtDNA CR (365 bp)**										

Eastern South Pacific (SP_{hist})	365	Tamura & Nei (gamma = 0.39)	This study	14	13	16 {2}	0.99 (0.03)	0.015 (0.008)	0.06 (0.54)	-10.5 (0.00)
Western South Atlantic (SA_{hist})	365	Tamura & Nei (gamma = 0.39)	This study	32	22	26 {12}	0.97 (0.02)	0.016 (0.008)	-0.55 (0.32)	-25.4 (0.00)
All historical SHFW combined	365	Tamura & Nei (gamma = 0.39)	This study	46	33	28	0.98 (0.01)	0.016 (0.008)	-0.52 (0.34)	-24.7 (0.00)

B) Genetic differentiation of *mtDNA CR* (365 bp)

	Eastern South Pacific (SP_{hist})	Western South Atlantic (SA_{hist})
Eastern South Pacific (SP_{hist})		0.02 (0.02)
Western South Atlantic (SA_{hist})	0.015 (0.20)	

Supplementary Table S7 Pairwise genetic differentiation oceanic comparison of historical and contemporary SHFW fin whale *mito* sequences. Φ_{ST} estimates are shown below the diagonal. Differences in haplotype frequencies (Fst) are shown above the diagonal. *p*-values denoting significance are shown in parentheses.

	Circumpolar (pre-1986)	Circumpolar (post-1986)
Circumpolar (pre-1986)		0.01 (0.4)
Circumpolar (post-1986)	-0.005 (0.8)	

Supplementary Table S8 Pairwise genetic differentiation oceanic comparison of historical and contemporary SHFW fin whale *mtDNA CR* sequences. Φ_{ST} estimates are shown below the diagonal. Differences in haplotype frequencies (Fst) are shown above the diagonal. *p*-values denoting significance are shown in parentheses.

	Circumpolar (pre-1986)	Circumpolar (post-1986)
Circumpolar (pre-1986)		0.02 (0.0)
Circumpolar (post-1986)	-0.003 (0.6)	

Supplementary Figure S1 Results from PMD tools *mapdamage*.

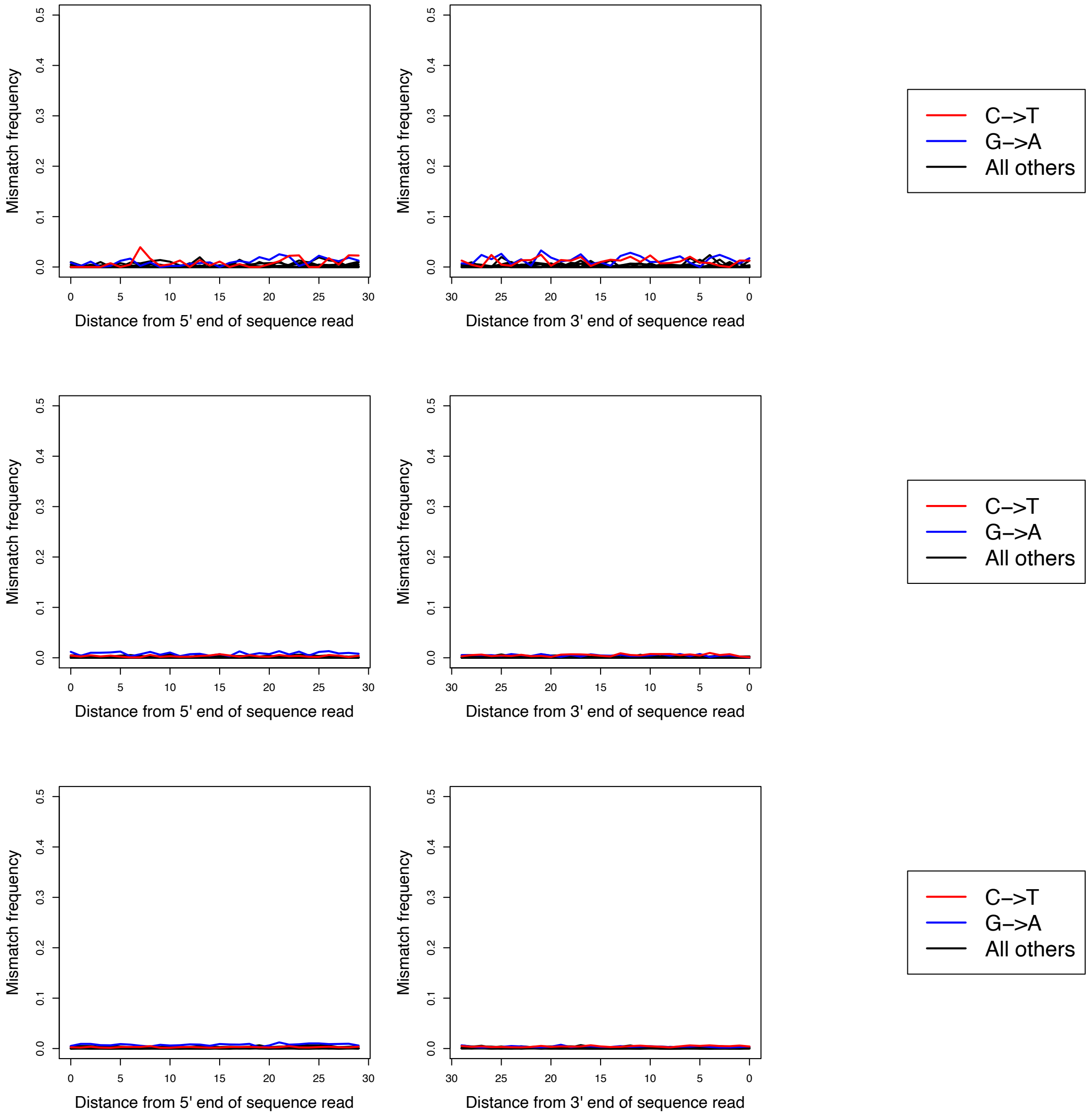

Supplementary Figure S2 Median-joining haplotype network of fin whale *mtDNA CR* sequences (241bp). Sequences originated from the North Pacific (oceanic only – NoGoC), the North Pacific (gulf of California only, GoC), North Atlantic (oceanic only), the Mediterranean, and the Southern Hemisphere.

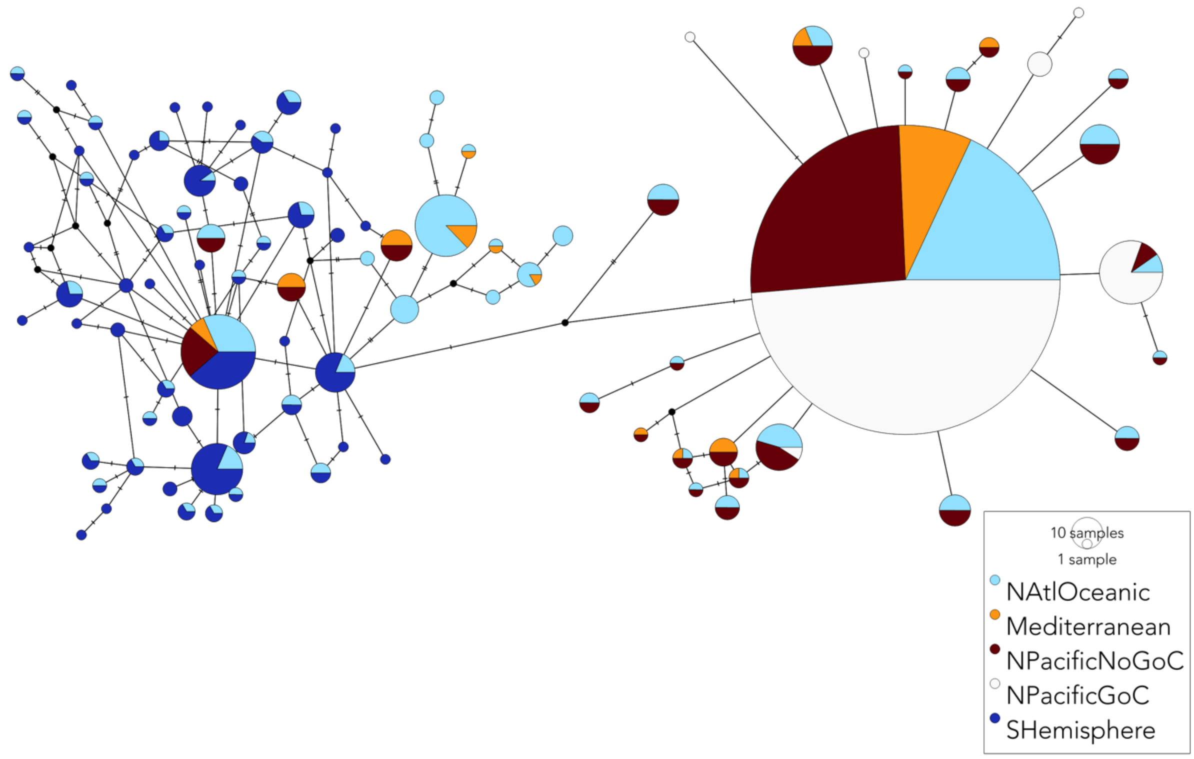

Supplementary Figure S3 MCMC tracer file for phylogenetic analysis performed in mrBayes.

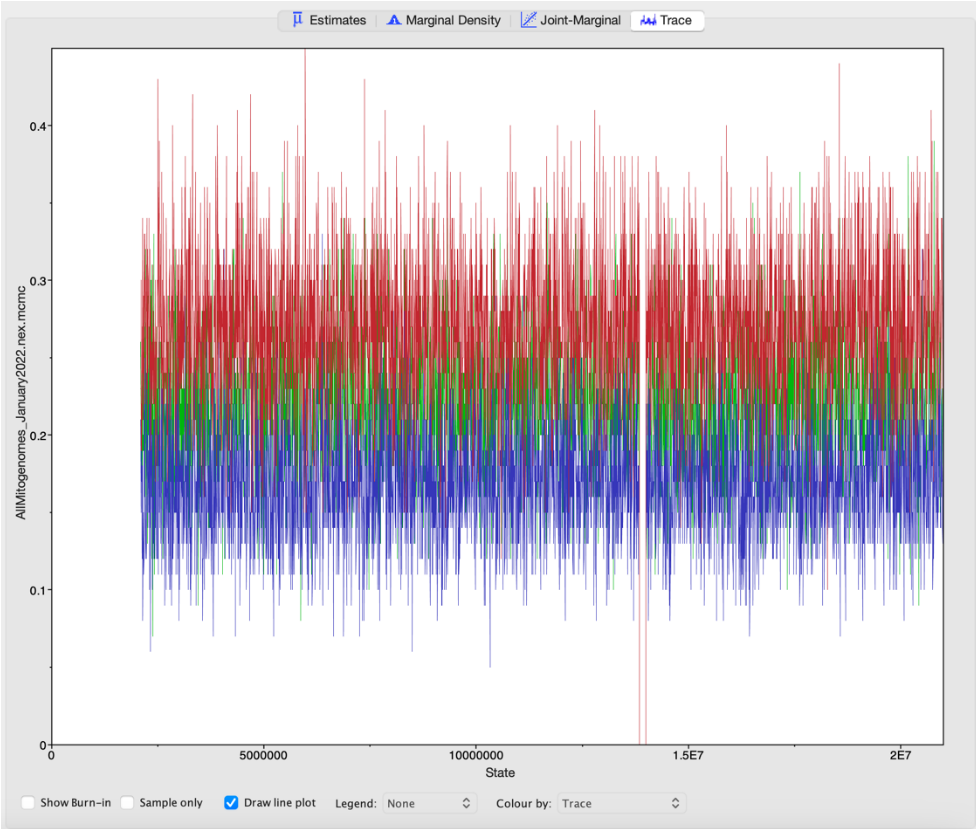

Supplementary Figure S4

Bayesian genealogy estimated using the fin whale mitogenomic sequences, coloured by SHFW sampling sites (grey – North Pacific; light blue – North Atlantic; black – humpback whale outgroup; dark red – eastern South Atlantic; dark gold – western South Atlantic; dark blue – Antarctic Peninsula and South Shetland Islands; green – low latitudes of South Pacific; teal – sub-Antarctic waters in the Indo-Pacific). [See separate file].

Supplementary Figure S5

Bayesian genealogy estimated using the fin whale mitogenomic sequences showing all posterior support values [See separate file].

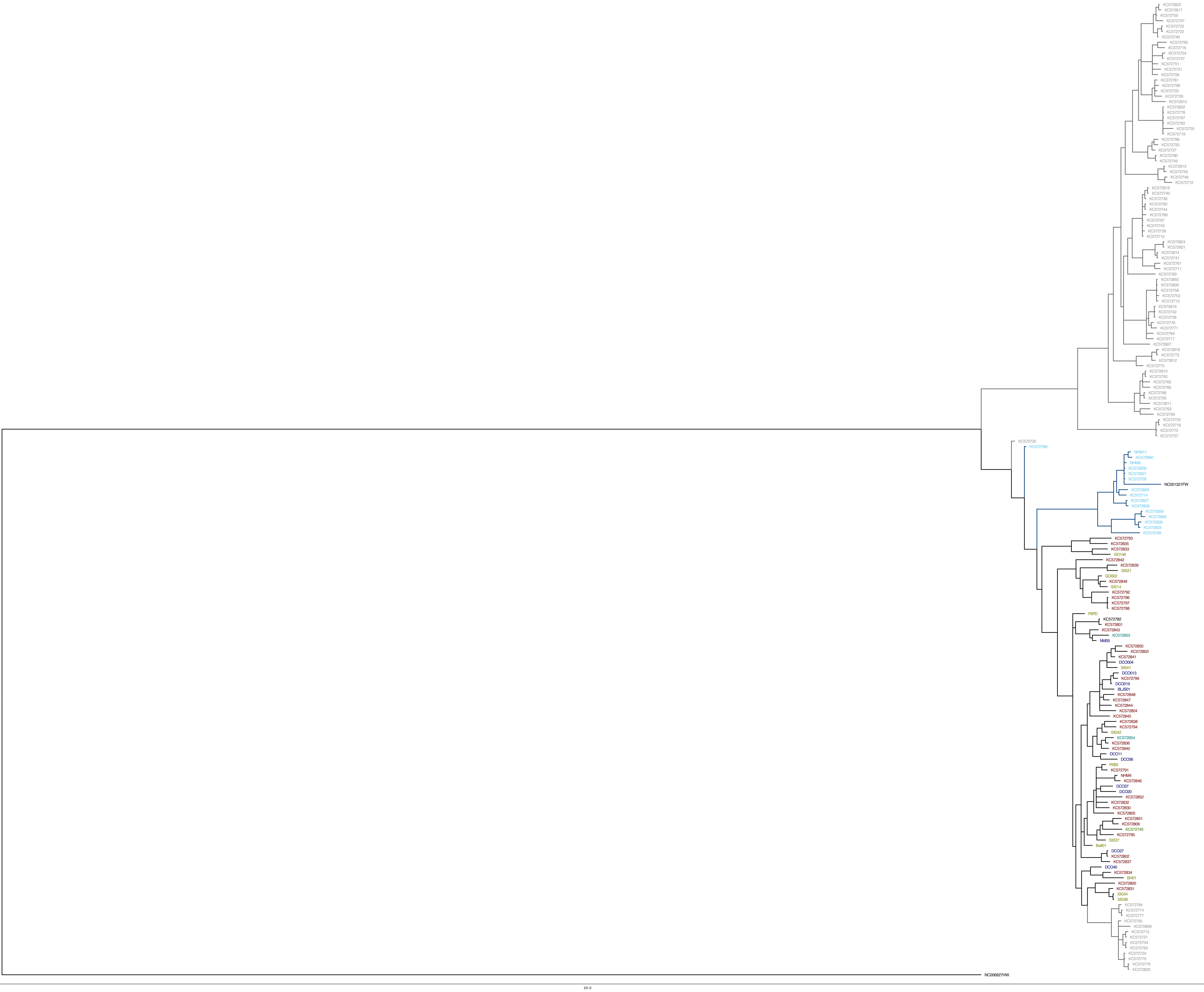

KC572823
KC572817
KC572755
KC572737
KC572723
KC572722
KC572746
KC572785
KC572716
KC572754
KC572747
KC572751
KC572731
KC572728
KC572781
KC572766
KC572733
KC572729
KC572815
KC572822
KC572778
KC572767
KC572762
KC572735
KC572719
KC572786
KC572720
KC572727
KC572780
KC572749
KC572813
KC572759
KC572748
KC572712
KC572819
KC572740
KC572738
KC572790
KC572744
KC572789
KC572787
KC572743
KC572739
KC572710
KC572824
KC572821
KC572814
KC572741
KC572761
KC572711
KC572769
KC572855
KC572809
KC572758
KC572753
KC572713
KC572816
KC572742
KC572736
KC572776
KC572771
KC572764
KC572717
KC572807
KC572818
KC572773
KC572812
KC572775
KC572810
KC572750
KC572765
KC572760
KC572768
KC572726
KC572811
KC572763
KC572756
KC572725
KC572718
KC572772
KC572757
KC572732
KC572788
NHM11
KC572860
NHM6
KC572858
KC572857
KC572709
NC001321FW
KC572829
KC572714
KC572827
KC572826
KC572859
KC572856
KC572828
KC572825
KC572708
KC572793
KC572835
KC572833
GO146
KC572842
KC572839
SI021
GO002
KC572849
SI014
KC572792
KC572796
KC572797
KC572798
FKPD
KC572782
KC572801
KC572843
KC572853
NMS5
KC572850
KC572803
KC572841
DCO004
SI041
DCO013
KC572799
DCO019
BLJS01
KC572848
KC572847
KC572844
KC572804
KC572845
KC572838
KC572794
SI042
KC572854
KC572836
KC572840
DCO11
DCO36
FKB3
KC572791
NHM4
KC572846
DCO37
DCO20
KC572852
KC572832
KC572830
KC572805
KC572851
KC572806
KC572745
KC572795
SI027
Ball01
DCO27
KC572802
KC572837
DCO40
KC572834
BH01
KC572800
KC572831
SI034
SI038
KC572784
KC572774
KC572777
KC572730
KC572808
KC572715
KC572721
KC572734
KC572783
KC572724
KC572770
KC572779
KC572820
NC006927HW
20.0

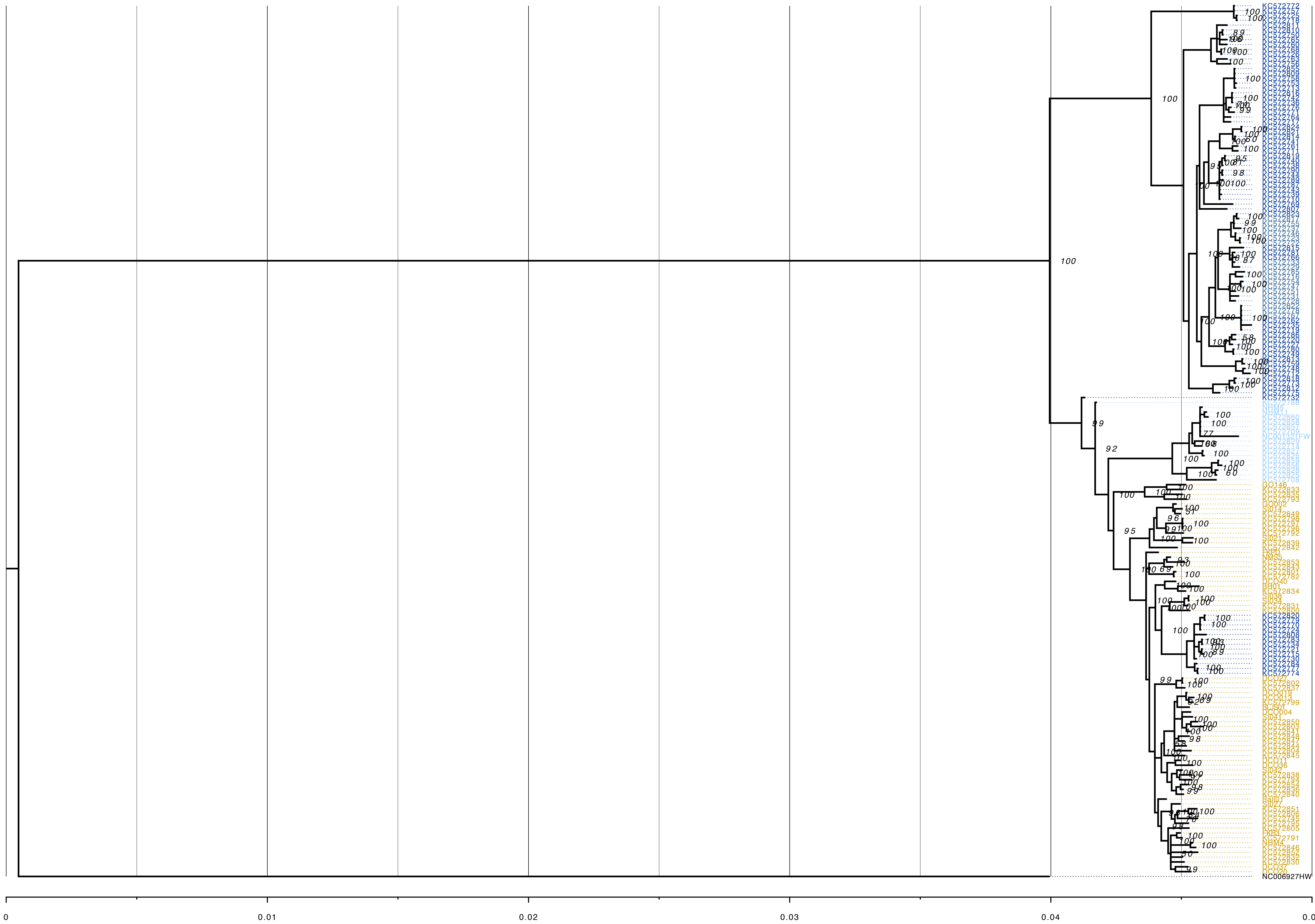
NC006927HW
0
0.01
0.02
0.03
0.04
